# Infanticide and Human Self Domestication

**DOI:** 10.3389/fpsyg.2021.667334

**Published:** 2021-05-05

**Authors:** Erik O. Kimbrough, Gordon M. Myers, Arthur J. Robson

**Affiliations:** ^1^Smith Institute for Political Economy & Philosophy, Chapman University, Orange, CA, United States; ^2^Department of Economics, Simon Fraser University, Burnaby, BC, Canada

**Keywords:** human self domestication, infanticide, pleistocene, reactive aggression, violence

## Introduction

The notion of domestication has played a role in attempts to understand human evolution going right back to Darwin (1859) (see Hare, 2017). Two of the leading current proponents of human self-domestication (HSD) are Hare and Wrangham. Hare (2017) lays out his account in “Survival of the Friendliest” and Wrangham has done much of the recent work on HSD; see Wrangham (2019) and references there.

HSD starts from the premise that our species' evolution in the later Pleistocene is consistent with a domestication syndrome, including traits such as a reduction in body mass, shortening of the face accompanied by a reduction in tooth size, reduced sexual dimorphism due to feminization, and a reduction in cranial capacity. Hare (2017 p. 157) argues that HSD, “draws on comparative, developmental, fossil, and neurobiological evidence to show that late human evolution was dominated by selection for intragroup prosociality over aggression. As a result, modern humans possess traits consistent with the syndrome associated with domestication in other animals (Table 1). An example is the long running silver fox experiments in Siberia.”

Sánchez-Villagra and van Schaik (2019) review the case for and against HSD. They accept some of the evidence but question the underlying theory. They make the important distinction between slow moving unintentional “domestication” and directional and intentional “selective breeding” to generate “improvement traits” as in the silver fox experiments. They argue that HSD as currently formulated is about unintentional domestication rather than selective breeding, and so using evidence for HSD from dog breeding, modern farm animals, and the silver fox data is misleading.

Wrangham (2019) is focused on possible underlying hypotheses for HSD and, rather than “survival of the friendliest,” he emphasizes selection against reactive aggression as key to human evolution and HSD.^1^ According to Wrangham, “To account for the domestication syndrome, proposals must explain what led to a decline in fitness of highly aggressive males, and why the explanatory factor applies only to H. sapiens and not to other species of Homo (Wrangham, 2019, abstract).”

He presents nine alternative hypotheses which could underly selection against reactive aggression: genetic group selection; group-structured cultural selection; social selection by female mate choice; social selection by choice of cooperative task partners; self-control; cooperative breeding; population density; use of lethal weapons; and language based conspiracy. The paper considers strengths and weaknesses of the nine. His favored hypothesis is the last—

“I conclude that the evolution of language-based conspiracy, which is a form of collective intentionality, was the key factor initiating and maintaining self-domestication in H. sapiens, because it is the most convincing mechanism for explaining the selective pressure against individually powerful fighters. Sophisticated language enabled males of low fighting prowess to cooperatively plan the execution of physically aggressive and domineering alpha males.” Wrangham (2019, abstract).

As Wrangham acknowledges, this hypothesis is not without weaknesses. For example; why is a coalition of violent dominant males limited to a one-person coalition? If a coalition of one aggressive male was not enough to control a band, why not a coalition of a few violent “brothers” to watch each other's backs and suppress the remaining band members? This possibility is heightened by Homo sapiens's capacity for proactive aggression (see Wrangham, 2018).^2^

Second, Wrangham offers that the timing of the evolution of cooperative communication is uncertain and so is a potential problem for his hypothesis. Specifically, Progovac and Benitez-Burraco (2019, 2020) argue that up to 200,000 years ago Homo sapiens' language likely consisted of not more than two-word crude compounds (their stage 2). These authors argue that language and HSD evolved together—that there were mutually reinforcing feedback loops between language evolution and HSD. This is inconsistent with sophisticated language initiating HSD.

As noted by Wrangham (2019 p. 9), “An alternative proposal for language evolution is that linguistic ability increased significantly only after the self-domestication process had been initiated (Hare, 2017). In that case, the language-based conspiracy hypothesis would be inadequate for explaining the origin of H. sapiens, and a different stimulus would be needed to account for the early stages of self-domestication.”

## Our Hypothesis

Our hypothesis, which is largely complementary to Wrangham, is that band elders engaged in infanticide and direct and indirect child homicide against the offspring of reactive aggressive adults through decisions during the foraging period of the Middle and Upper Pleistocene. We hypothesize that elders may have targeted the offspring of reactively aggressive males (and females) as retaliation for behaviors that were not good for the elders or their offspring and because surreptitiously killing the offspring of violent males was much less dangerous to the elders than killing the violent males. Such retaliation could have selected against reactive aggression as a genetic consequence. In other words, infanticide could have been Wrangham's “different stimulus” initiating HSD. Our argument is that the earliest language of single words (kill), and certainly the crude compounds, “kill-baby or “like father” and “like son,” would be enough to organize the “execution of an infant” in a relatively secluded birthing site. Infanticide effectively becomes a second moment of mate choice. Such an action could have been relatively safely concealed since an infant dying in childbirth in forager socio-ecological conditions was likely not unusual (see below). The relative simplicity of the language capacities necessary for infanticide contrasts with the more sophisticated language necessary to organize a safe coup and execution of a reactively aggressive alpha male.

If infanticide was employed in a way that selected for a reduction in reactive aggression (e.g., selectively targeting the offspring of male rapists or physically abusive mates), then it would have contributed to self-domestication. As in Progovac and Benitez-Burraco (2019, 2020) this could then trigger the evolution of more sophisticated language. Eventually complex language may have not only made possible the safe execution of a reactively aggressive adult male, but also may have helped establish a norm of mutual agreement for mate choice—rules against rape that are enforced by a coalition, as argued by Wrangham (2019).

Finally, a factor potentially unique to Homo sapiens would be the sapience to recognize the transmission of traits (e.g., appearance) and behaviors (e.g., reactive violence) from parents to offspring.^3^ If this recognition were present along with sophisticated communication and a capacity for proactive aggression, then selection against reactive aggression could have been the intended direct selection of a trait, rendering HSD more consistent with selective breeding and thereby readmitting comparison with data such as that produced by the silver fox experiments.

## Evidence On Infanticide And Child Homicide In The Pleistocene

Empirical evidence of infanticide has been documented in both human societies and in non-humans. See Harris (1977 chapter 13) for data on indirect infanticide in modern societies and Hausfater and Hrdy (1984) on infanticide in animals and humans. See Scrimshaw (1984), on infanticide in human societies across the globe.

What is the evidence for the Pleistocene? Renfrew and Bahn (2000) report a world population in the 5–20 million range at the end of the long Pleistocene. Cohen (1980 p. 275) estimates annual population growth of 0.001–0.003% during the Pleistocene with a hundred-fold increase during the Holocene and a 1,000-fold increase in modern times. Li and Durbin (2011) use whole-genome DNA sequencing of modern individuals to estimate effective Homo sapiens population size throughout the Pleistocene. They find population increased to a peak 200–100k years ago. This growth was followed by a drop until it began to increase again 50–30k years ago, followed by a definite increase in the late Pleistocene with rapid increase during the Mesolithic. Further, it is usually argued that the quality of life as a forager was higher than for the early sedentary “agriculturists” of the Holocene.^4^ Klein and Steele (2013) provide evidence of early behavioral modernism and a “rich life” which was achieved at low population densities in, for example, the Blombos cave 70,000 years ago. It seems they avoided the Malthusian trap through population control.

There is much agreement among anthropologists and archaeologists that Pleistocene peoples controlled their population size (see Birdsell, 1968; Harris, 1977, chapter 2; Cohen, 1980; Hassan, 1980; Lee, 1980; Ripley, 1980; Hausfater and Hrdy, 1984; Harris, 1993, chapter 13; Megarry, 1995, p. 221), and they did so without access to contraception or safe medical abortion. Instead, it seems the major mechanisms would have been culturally demanded abstinence, disruption of the menstrual cycle through extended years of breast-feeding, unsafe abortion, direct and indirect infanticide, and direct and indirect child homicide.

Among modern foragers, levels of infanticide have been estimated as high as 15–50%. The differential incidence of this resulted in a male-female population ratio of 1.3 or higher (Birdsell, 1968, p. 236 and 243). Hassan (1975) suggests percentages in the 23–35% range for infanticide and abortion. Hill and Hurtado (2017 p. 168, 400, and 449) in their careful study of Ache bands report that infanticide and homicide were the most common cause of death of unweaned infants. They also report child homicide (before age of 10) of one form or another in the pre-contact forest period of 14% for males and 23% for females.

While there is evidence that foraging societies exhibited significant levels of violent death, due to inter-band violence (warfare, raiding, club fighting, etc.), Hill and Hurtado (2017 p. 173, Table 5.1, p. 171–173) report intra-band homicide was relatively uncommon after childhood. Of the 87 deaths of adult males age 15–54 during the 80-year forest period only one (a teenager) was listed as a homicide by an own band member. Further, the two adult males with the most surviving offspring were purportedly not dominant-type males, rather they were hard working, peaceful men, who were good to women (Hill and Hurtado, 2017, p. 389–391).

## Infanticide And Homicide As Social Choices

These decisions and actions were not private to the parents and so did not need to be taken in the interest of the parents. A Homo sapiens mother often requires assistance with birth due to the bent birth canal. This makes it dangerous for a mother to isolate herself during birth.^5^ Birthing sites within an Ache band involved men and women [often not the likely father, Hill and Hurtado (2017) (p. 254)], and with the !Kung and the Ayoreo bands it was women only at the birthing sites (Ripley, 1980; Bugos and McCarthy, 1984). In a band structure, there were also many opportunities and evidence for direct and indirect child homicide through a lack of necessary care or abandonment in the mobile lifestyle of foragers.

Given the importance of others (elders) in these decisions we ask what is in the interest of the elder. Meat sharing is considered an almost defining characteristic of a band. Given meat sharing, environmental capacity constraints, and a large potential capacity for human population growth, controlling overall band population would be good for the elder. Imagine then that an elder intends to eliminate 20% of births to maintain a stable population; which 20% would the elder choose? Focusing population control on female children could make sense as women were usually not meat producers and the number of females is a population control instrument in a non-monogamous society. Cashdan (1989) pointed out that among the !Kung, good hunters had more children and more children who reached maturity. Given a desired population size and meat sharing, if good hunters had more children reach maturity it likely means that children of low productivity hunters are being selected against. Meat sharing would also rationalize an elder targeting “expensive” children: premature; weak; and orphaned children (Daly and Wilson, 1988). Hill and Hurtado (2017) p. 434–439 note all of these cases among the Ache. Further, if the physically weak are disproportionately targeted what about those prone to psychological disturbance, for example, the children of reactive aggressive adults? Scrimshaw (1984 Table 1, p. 445) in regard to over 100 societies undertaking infanticide, includes the cases above but also lists: mentally incapable mothers; disliked or unattractive infants and children; and the child of a hated father.

As noted above, one important domestication syndrome trait was face shortening. Homo sapiens faces shortened through the foraging period and then started lengthening in the Holocene (Hare, 2017, p. 168). This is important evidence consistent with our hypothesis as population control and so infanticide would have played a lesser role in the Holocene.

One of the interesting and difficult to explain complexities of HSD is the evidence of simultaneous selection against reactive aggression and selection for proactive aggression. While elders would have found reactive aggression almost certainly unattractive, they may well have found proactive aggression desirable, if it could be channeled to the defense of the band. In fact, possibly the most attractive trait for an elder would be a propensity for rule following (which facilitates acquisition of and conformity to group norms). Elders would be prone to favor individuals who listen to their elders. By contrast to selection for a particular tendency expressed in general and independent of context, selection for rule-following is better for elders because it implies behavioral plasticity. That is, it allows the right behavior to be contingent on the situation.^6^ See Kimbrough and Vostroknutov (2016, 2018) for individually irrational rule following in a decision theoretical experiment. Both Hare and Wrangham provide some support for self-control and norms as elements in HSD.

In the end, the evidence we have on the role for infanticide is largely circumstantial. We now suggest some tests which would provide direct evidence. Unbalanced sex ratios in children vs. adult paleolithic skeletons would provide strong evidence for infanticide. The gold standard for our hypothesis, would be finding direct genetic evidence of reactive aggression in higher proportions in paleolithic infant skeletons than in paleolithic adult skeletons. For a discussion of recent work on genes associated with reactive aggression see Zhang et al. (2016). Further, there are various conditions which lead to reactive aggression. One is bipolar disorder, which likely has a genetic component. Somewhat less direct, but potentially interesting is the following. If infanticide is largely a forager phenomenon, we would expect a negative relationship between the amount of time that any population's ancestors have been engaged in agriculture and the degree of HSD (e.g., less facial lengthening and more genes associated with reactive aggression in descendants of long-time farming populations). Testing of such effects seems feasible.

## Discussion

We have argued that infanticide is not uncommon in many human societies and that it may have been a key trigger in initiating the HSD process. We argued that very simple and early language (crude compounds), would be enough to organize a rather private infanticide. The relative simplicity of the language capacities necessary for infanticide contrasts with the more sophisticated language necessary to organize a safe coup and execution of a reactively aggressive alpha male. If infanticide was employed in a way that selected for a reduction in impulsive or reactive violence (e.g., selectively targeting the offspring of a male rapist or an abusive partner), then it would have contributed to self-domestication. This could then trigger the evolution of more sophisticated forms of language and more HSD as in the work of Progovac and Benitez-Burraco (2019, 2020). Eventually complex language would have enabled the safe execution of a reactively aggressive adult male as in Wrangham (2019), or female mate choice enforced by a coalition. One way of interpreting our hypothesis is that infanticide was a tool that provided females (and those elders who were involved in childbirth) an additional degree of control over reproduction and that utilizing that control to reduce the fitness of rapists and other aggressors was key to the evolution of modern humans.

## Author Contributions

All authors listed have made a substantial, direct and intellectual contribution to the work, and approved it for publication.

## Conflict of Interest

The authors declare that the research was conducted in the absence of any commercial or financial relationships that could be construed as a potential conflict of interest.

## Footnotes

^1^Reactive aggression is similar to “impulsive, affective, hostile, defensive, emotional, or hot aggression,” and it is contrasted with proactive aggression, which is also known as “instrumental, predatory, offensive, controlled or cold aggression” (Wrangham, 2018, p. 246).

^2^Gintis et al. (2015, p. 331) labels Wrangham's coalition type a “leveling coalition.” They label a coalition type supporting an alpha male as a “rank changing” coalition. Hare (2017) provides an example of female bonobos forming a cooperative coalition against a violent sexually aggressive male and so selecting against violent males. This argument is based on earlier work by Hare et al. (2012).

^3^When Homo sapiens and, for example, Neanderthals were interbreeding, 65–47kya, this may have become obvious.

^4^Angel (1975) studying skeletal remains from the upper Pleistocene found that these people grew taller and had less tooth loss than all but the most recent humans. Renfrew and Bahn (2000 p.452), commenting on a number of studies, conclude that there seems to be a pattern of reduced mechanical stress (injuries), but increased infections and nutritional stress with the adoption of early agriculture. Workloads were also likely not severe. Cashdan (1989) points to some variability across different modern foraging groups, but suggests a work week similar to our own (p. 26).

^5^Further, the objections of a mother may have been mitigated by women in foraging societies having an incentive to have few dependent children because of the problem, if not impossibility, of carrying all possessions, gathered food and more than one child over the great distances travelled in a foraging lifestyle (Lee, 1980). This explanation and the increase in the productive value of children as labor in early agriculture are also consistent with the dramatic increases in population in the Holocene.

^6^This is also consistent with the view offered by Chudek and Henrich (2011) since a capacity to learn and follow group rules is an important underpinning of theories of cumulative culture. Infanticide of the kind we describe could have been an important selection pressure in the evolution of a “norm psychology”.
